# Validation of a metabolite–GWAS network for *Populus trichocarpa* family 1 UDP-glycosyltransferases

**DOI:** 10.3389/fpls.2023.1210146

**Published:** 2023-07-21

**Authors:** Patricia M. B. Saint-Vincent, Anna Furches, Stephanie Galanie, Erica Teixeira Prates, Jessa L. Aldridge, Audrey Labbe, Nan Zhao, Madhavi Z. Martin, Priya Ranjan, Piet Jones, David Kainer, Udaya C. Kalluri, Jin-Gui Chen, Wellington Muchero, Daniel A. Jacobson, Timothy J. Tschaplinski

**Affiliations:** ^1^ Center for Bioenergy Innovation, Biosciences Division, Oak Ridge National Laboratory, Oak Ridge, TN, United States; ^2^ Bredesen Center for Interdisciplinary Research, University of Tennessee, Knoxville, TN, United States; ^3^ Protein Engineering, Merck & Co., Inc., Rahway, NJ, United States; ^4^ Department of Biomedical Sciences, Quillen College of Medicine, East Tennessee State University, Johnson City, TN, United States; ^5^ School of Electrical Engineering, Southeast University, Nanjing, China

**Keywords:** glycosyltransferase, *Populus*, functional genomics, high throughput, GWAS, metabolite-gene validation, metabolomics

## Abstract

Metabolite genome-wide association studies (mGWASs) are increasingly used to discover the genetic basis of target phenotypes in plants such as *Populus trichocarpa*, a biofuel feedstock and model woody plant species. Despite their growing importance in plant genetics and metabolomics, few mGWASs are experimentally validated. Here, we present a functional genomics workflow for validating mGWAS-predicted enzyme–substrate relationships. We focus on uridine diphosphate–glycosyltransferases (UGTs), a large family of enzymes that catalyze sugar transfer to a variety of plant secondary metabolites involved in defense, signaling, and lignification. Glycosylation influences physiological roles, localization within cells and tissues, and metabolic fates of these metabolites. UGTs have substantially expanded in *P. trichocarpa*, presenting a challenge for large-scale characterization. Using a high-throughput assay, we produced substrate acceptance profiles for 40 previously uncharacterized candidate enzymes. Assays confirmed 10 of 13 leaf mGWAS associations, and a focused metabolite screen demonstrated varying levels of substrate specificity among UGTs. A substrate binding model case study of UGT-23 rationalized observed enzyme activities and mGWAS associations, including glycosylation of trichocarpinene to produce trichocarpin, a major higher-order salicylate in *P. trichocarpa.* We identified UGTs putatively involved in lignan, flavonoid, salicylate, and phytohormone metabolism, with potential implications for cell wall biosynthesis, nitrogen uptake, and biotic and abiotic stress response that determine sustainable biomass crop production. Our results provide new support for *in silico* analyses and evidence-based guidance for *in vivo* functional characterization.

## 1 Introduction

**Figure 1:**
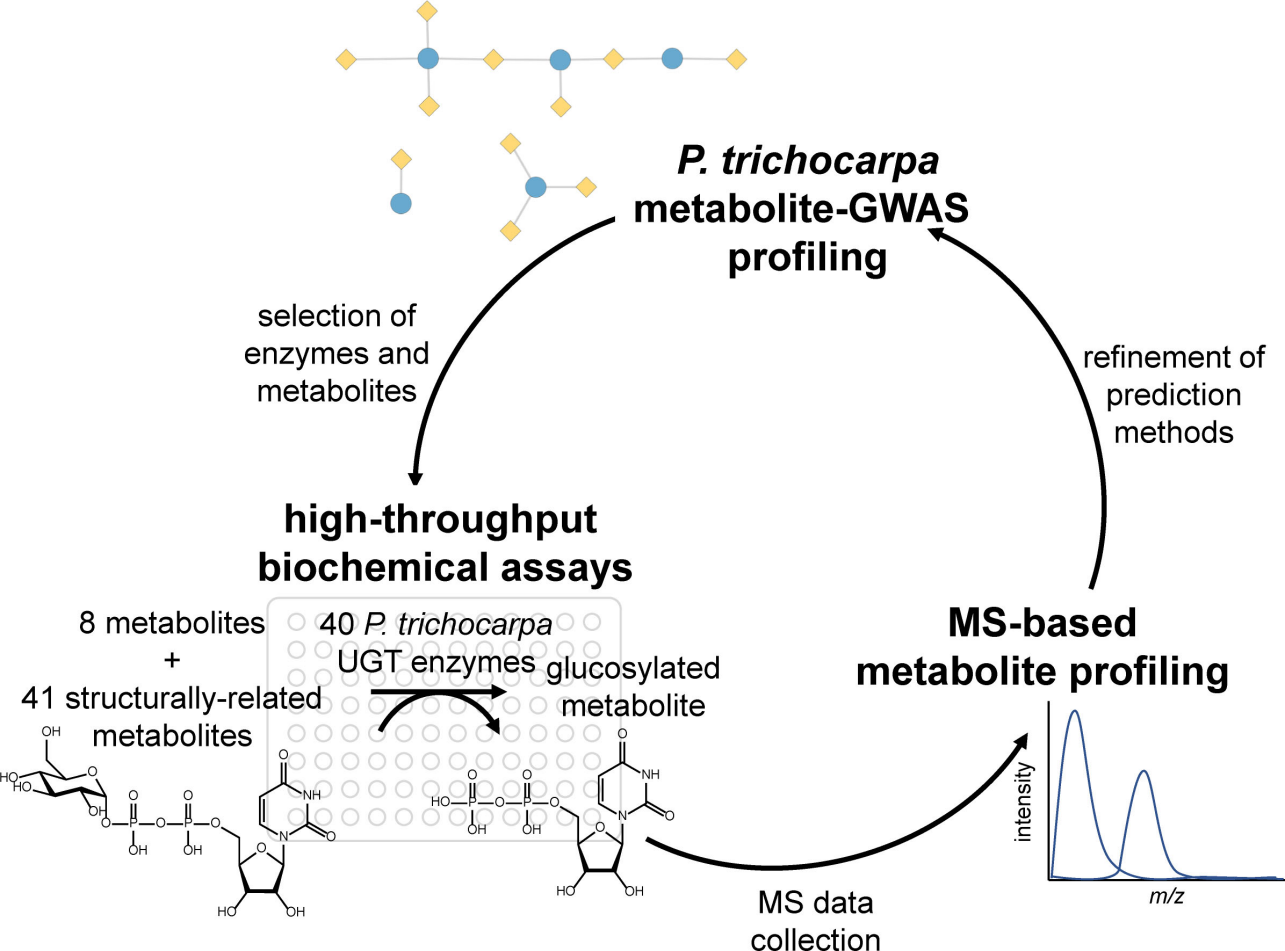
Project workflow, starting with metabolite–GWAS profiling to inform biochemical assays, which were analyzed using MS-based metabolite profiling. The results of the assays inform metabolite–GWAS prediction methods for future studies. GWAS, genome-wide association study.

Metabolite genome-wide association studies (mGWASs) are increasingly used in crop breeding and bioengineering programs to discover the genetic basis of target phenotypes (Ding et al., 2021). Secondary metabolites, which are involved in defense, signaling, and lignification, are well suited for mGWASs, because their biosynthesis is typically controlled by a few loci of large effect and is highly heritable (Fang and Luo, 2019). The use of mGWASs in systems biology studies on complex processes is particularly valuable, because metabolite phenotypes can be directly measured and reflect whole plant physiology and environmental interactions. For example, recent studies in the biofuel feedstock *Populus trichocarpa* incorporated mGWAS layers in multiomics analyses to identify candidate genes involved in cell wall biosynthesis and control of wood traits (Chhetri et al., 2019; Furches et al., 2019; Chhetri et al., 2020). However, despite their growing importance in plant studies, few mGWAS associations are experimentally validated. Here, we present a functional genomics workflow (**Figure 1**) for validating mGWAS predictions, focusing on uridine diphosphate (UDP)–glycosyltransferases (UGTs) in *P. trichocarpa* as a case study.

UGTs, the largest family of glycosyltransferases in the plant kingdom, catalyze sugar transfer to secondary metabolites. Sugar donors include UDP-glucose, UDP-rhamnose, UDP-glucuronic acid, UDP-galactose, and UDP-xylose (Louveau et al., 2018; Wang et al., 2019). The sugar moiety endows new functionality, directing metabolite localization within cells and tissues, altering physiological effects [e.g., via inactivation of signaling (Mhamdi, 2019)], influencing metabolic degradation (Bowles et al., 2006; Wang and Hou, 2009), and facilitating long-distance transport (Park et al., 2007). Given that UGTs directly affect the accumulation or degradation of metabolites in plant tissues, understanding their function in *Populus* is important for genotype selection aimed at improving biofuels, valorizing biomass, and providing targets for engineering sustainable metabolites (Salas and Mendez, 2007; Tsai et al., 2006; Payyavula et al., 2014; Tschaplinski et al., 2019). Previous studies in *Populus* showed that UGTs play critical roles in growth–defense trade-offs, including response to salt, drought (Tschaplinski et al., 2019; Rehman et al., 2022), and herbivory (Babst et al., 2010; Fellenberg et al., 2020). UGT activities also aid in the determination of leaf and stomatal morphology (Chhetri et al., 2019; Chhetri et al., 2020). However, many *Populus* UGTs have yet to be characterized, and most have not been experimentally validated. Characterization of this large enzyme family presents a challenge due to expansion in land plants and extensive duplication within the *Populus* genome (Geisler-Lee et al., 2006; Yonekura-Sakikibara and Hanada, 2011; Caputi et al., 2012; Wilson and Tan, 2019).

Here, we validate mGWAS predicted substrates through the expression of synthetic codon-optimized genes, high-throughput biochemical assays, and mass spectrometry (MS)-based analysis to generate metabolic profiles **Figure 1**. First, mGWASs were used to detect UGT–metabolite associations. Next, associated metabolites were used to create a substrate panel for testing the activities of a subset of UGTs. Enzymes were expressed *in vitro* and assayed against predicted metabolites; confirmed interactions were compared to the mGWAS network. Lastly, virtual screening predicted the relative binding affinities of different compounds and rationalized observed enzyme activities and mGWAS associations. Results confirmed mGWAS associations and revealed varying levels of substrate specificity among UGT candidates.

## 2 Methods

### 2.1 Sample collection, metabolomics analysis, and mGWAS

Detailed methods for sample collection, metabolite extraction, and gas chromatography–mass spectrometry (GC-MS) analysis were previously described (Weighill et al., 2018). Briefly, leaf tissue was collected in July 2012 from 851 unique *P. trichocarpa* genotypes in a common garden in Clatskanie, Oregon (Weighill et al., 2018), established from wild accessions in the native range in the Pacific Northwest (Chhetri et al., 2020). Samples were flash frozen on dry ice in liquid N_2_ and stored at −80 °C until analysis. Metabolites were extracted from pulverized freeze-dried leaves in 80% ethanol with sorbitol as an internal standard, converted to trimethylsilyl (TMS) derivatives, and characterized using GC-MS as described previously (Tschaplinski et al., 2012; Weighill et al., 2018). Full metabolite profiles were collected for each genotype, and outlier metabolite peaks greater than six median absolute deviations (MADs) from the population median were removed, resulting in 818 metabolomics phenotypes.

### 2.2 mGWAS

The mGWAS has been reported and described previously (Chhetri et al., 2020). Briefly, single-nucleotide polymorphism (SNP) data from 869 whole genome resequenced *P. trichocarpa* trees were utilized after removing closely related and highly differentiated genotypes and SNPs with minor allele frequency (MAF) <0.01 and population call rate >0.75 (Chhetri et al., 2020). mGWAS was performed using a linkage disequilibrium pruned genomic relationship matrix and the linear mixed model (LMM) implemented in EMMAX (Kang et al., 2010) with ADIOS v1.13 (Lofstead et al., 2008) for scaling (see Furches et al., 2019). *p*-Values were corrected for multiple testing (Benjamini & Hochberg, 1995) using a false discovery rate (FDR) threshold of 0.1 (*p*_(_ *_i_*_)_ ≤ (*i*/*m*) * *Q*, where *i* is the rank of *p*-value, *m* is the number of SNPs = 8,238,357, and *Q* is the FDR threshold = 0.1).

A second analysis was performed to identify associations with rare SNPs (MAF < 0.01). SNPs located within gene boundaries or in 2-kb flanking regions were grouped as a single region and analyzed jointly (Furches et al., 2019). The Sequence Kernel Association Test implemented in RVtest (Zhan et al., 2016) was performed on each annotated gene region (41,335 in *P. trichocarpa* v3.0). With the use of LMM, combined region scores were created in which component SNPs were MAF-weighted (beta distribution shape parameters: 1, 25). *p*-Values were corrected for multiple testing using an FDR of 0.1 (*p*_(_ *_i_*_)_ ≤ (*i*/*m*) * *Q*, where *i* is the rank of *p*-value, *m* is the number of gene regions = 41,335, and *Q* is the FDR threshold = 0.1).

A third analysis on 1,254 genotypes was performed (Zhang et al., 2018) after conducting a SnpEff analysis and filtering for SNPs with MAF > 0.05 using the Efficient Mixed-Model Association algorithm implemented in EMMAX with kinship as the correction factor for genetic background effects (Zhou and Stephens, 2012). A *p*-value threshold of 6.1 × 10^−09^ (0.05/8,253,066) was used to determine significance using the Bonferroni correction for multiple testing.

In all analyses, SNPs were mapped to the genes in which they were located or to the nearest neighboring gene. The *P. trichocarpa* genome sequence, annotation data, and Gene Atlas expression data are available at https://phytozome-next.jgi.doe.gov/. *P. trichocarpa* SNP and indel data are available at https://cbi.ornl.gov/gwas-dataset/.

### 2.3 Network analysis and candidate gene selection

mGWAS networks were created using Python (v3.7.3) and merged on *P. trichocarpa* gene and metabolite identifiers (nodes) and mGWAS associations (edges). Gene annotations from Phytozome (https://phytozome-next.jgi.doe.gov/) and MapMan Mercator pipeline (Goodstein et al., 2012; Schwacke et al., 2019) were incorporated into network metadata. The merged network was filtered to include genes containing PFAM UDPGT (PF00201: uridine 5′-diphospho-glucuronosyltransferase) protein domains (https://pfam.xfam.org/; Mistry et al., 2021) and full-length open reading frames (ORFs). The network was curated based on sequence homology and network-based Jaccard index to down-select for unique candidates and was visualized using Cytoscape v3.7.1 (http://cytoscape.org; Shannon et al., 2003).

### 2.4 Phylogenetic tree and sequence similarity network

Full-length amino acid sequences were aligned using Clustal Omega default settings (https://www.ebi.ac.uk/Tools/msa/clustalo/), and a phylogenetic tree was constructed using EMBL-EBI (https://www.ebi.ac.uk/Tools/phylogeny/simple_phylogeny/) with neighbor-joining clustering method, no distance correction, and no gap exclusion. The phylogenetic tree was visualized in the Interactive Tree of Life (https://itol.embl.de/) in rectangular mode with branch lengths displayed. The Enzyme Function Initiative Enzyme Similarity Tool (EFI-EST, https://efi.igb.illinois.edu/efi-est/tutorial.php) was used to make the sequence similarity network (SSN), visualized using Cytoscape.

### 2.5 Candidate gene transcription

To characterize the tissue-specific expression of candidates, *P. trichocarpa* reference Nisqually-1 RNA-seq data was obtained from the DOE Joint Genome Institute (JGI) Plant Gene Atlas (https://phytozome.jgi.doe.gov/pz/portal.html) for leaf, stem, root, and bud tissues at multiple developmental stages. RNA-seq read trimming, alignment, and transcripts per million (TPM) calculations were described in Furches et al. (2019). Six outliers were removed that were inconsistent with tissue type and treatment subgroups. Replicates were averaged, and a clustered heatmap was created using Seaborn v0.11.1 (https://seaborn.pydata.org/index.html) with Euclidean distance metric, Ward clustering method, and normalization across tissues.

To characterize population variation in candidate expression, *P. trichocarpa* leaf, xylem, and root RNA-seq data collected from a common garden were obtained from the National Center for Biotechnology Information Sequence Read Archive (NCBI SRA) database (www.ncbi.nlm.nih.gov/sra; see Yates et al., 2021, Table S11, for SRA identifiers). Tissue collection and processing were described by Zhang et al. (2018) and Yates et al. (2021). Data processing and TPM calculations were described by Furches et al. (2019). Outliers were removed using a MAD threshold of seven. Clustered heatmaps were created using Seaborn with Euclidean distance metric, Ward clustering method, and normalization across samples.

### 2.6 Gene synthesis and expression

Media and buffer components were purchased from Sigma-Aldrich (St. Louis, MO, USA). Genes were codon-optimized and synthesized in the pQE-60 vector (Qiagen, Hilden, Germany) by Biomatik for expression in NEB^®^ Express *I* ^q^ *Escherichia coli* (**Table S1**). Transformants were incubated at 37 °C and 200 rpm shaking for 18 h (180 µl of Luria-Bertabi (LB), 100 µg/ml of ampicillin, and 1% *v*/*v* glucose). Aliquots (20 µl) of overnight cultures were diluted into terrific broth (380 µl and 100 µg/ml of ampicillin) and grown to an optical density (OD) of 0.8 at 30 °C with 300 rpm shaking. Protein expression was induced with 10 µl of 40 mM isopropyl β-d-1-thiogalactopyranoside followed by incubation at 30 °C with 300 rpm shaking for 19 h. Cells were harvested by centrifugation (4 °C, 4,750 rpm, 10 min), resuspended in 200 µl of lysis buffer (20 mM of Tris (pH 8.0), 0.1 mM of CaCl_2_, 2.5 mM of MgCl_2_, 1 mg/ml of lysozyme, 1 mg/ml of polymyxin B sulfate, and DNase I), and gently shaken at room temperature (rt) for 2 h. Insoluble cell material was removed by centrifugation (4 °C, 4,750 rpm, 20 min).

### 2.7 Glycosylation

Substrates and buffer components were purchased from Sigma-Aldrich. Reaction mixtures were prepared (1 mM of substrate, 5 mM of UDP-glucose, and 20 mM of Tris-HCl, pH 7.5). Aliquots (30 µl) of the reaction mixture were added to 10 µl of UGT lysate in 96-well plates and incubated at rt for 18 h with gentle shaking. Reactions were quenched by diluting 20 µl of aliquots into 180 µl 50% *v*/*v* acetonitrile (0.1% *v*/*v* formic acid). After brief vortexing and centrifugation (4 °C, 4,750 rpm, 10 min), supernatants were diluted 1:4 into liquid chromatography–mass spectrometry (LC-MS)-grade water for analysis. For quality control, buffer-only, enzyme-only, and substrate-only reactions for each enzyme and each substrate were also analyzed.

### 2.8 High-throughput detection of enzyme activity

Multiple reaction monitoring (MRM) methods were built for each substrate, UDP-glc, and UDP by optimizing fragmentor voltages and collision energies for the substrate and applying these parameters to define transitions (confirmation peaks) for the glucose conjugate(s) (**Table S5**). A water–acetonitrile (0.1% *v*/*v* formic acid) gradient was run with an Agilent (Santa Clara, CA, USA) Zorbax Eclipse Plus C_18_ 50 × 2.1 mm column (1.8-µm particle size) on an Agilent 6470 QQQ MS/MS with a 1260 Prime ultrahigh-performance liquid chromatograph. Instrument tuning was checked daily using LC/MS Tuning Solution for electrospray ionization (ESI) (Agilent). The gradient method was adjusted for each substrate to ensure observed glucose conjugates eluted with k > 0.5 from the solvent front (**Table S6**). Data were analyzed with MassHunter Quantitative Analysis B.09, and enzyme activity was recorded for a substrate if the product peak signal-to-noise ratio was >3 and a product confirmation peak was detected. MRM data are provided in **Table S7**.

### 2.9 Virtual screening

**Figure 2:**
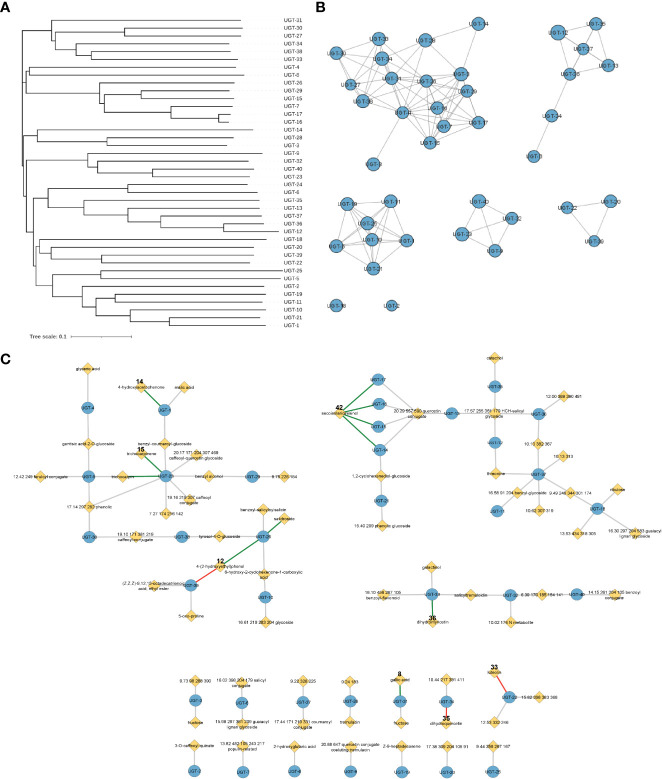
Representations of enzyme and metabolite–enzyme relationships. **(A)** Phylogenetic tree and **(B)** sequence similarity network with an e-value of −60 of the 40 *Populus trichocarpa* UGTs in this study. Diameter of the circle in panel B is proportional to the length of the protein sequence. **(C)** Associations of 40 UGT genes with *P. trichocarpa* leaf metabolites in a metabolic genome-wide association study (GWAS). Genes are indicated by blue circles, and metabolites are indicated by yellow diamonds. Gray lines indicate connections predicted by the GWAS analysis with an FDR-corrected *p*-value <0.1, green lines indicate GWAS connections that were confirmed in biochemical assays, and red lines indicate that biochemical assays did not support the predicted metabolite–GWAS relationship. Gallic acid (8) and dihydromyricetin (36) connections were validated by Glc-3 products. The trichocarpin and UGT-23 connection was inferred by observation of the diglucoside product of 15. FDR, false discovery rate.

Virtual screening to predict relative binding affinities of substrates used UGT-23, the structure of which was predicted with AlphaFold (Jumper et al., 2021), and experimentally validated substrates as a case study. The average predicted local distance difference test (pLDDT) of 91.5 was obtained for the model, indicating high accuracy (Mariani et al., 2013). The initial conformers of trichocarpinene (15), trichocarpin, and 12 other compounds (**Figure 2B**) were generated using RDKit (RDKit: Open-source cheminformatics; https://www.rdkit.org).

The positions of the side chains of a few amino acid residues in the substrate binding site were manually modified using the structure of the glycosyltransferase UGT78G1 bound to myricetin and UDP (PDB_ID: 3HBF), as it is considered to be representative of the ligand-bound conformation of UGTs (Modolo et al., 2009). UDP was added to the model using its coordinates in the template. The modified structure bound to UDP was relaxed via a short (50 ps) molecular dynamics simulation using GROMACS-2019 (Abraham et al., 2015). The CHARMM36 force field (Hart et al., 2012; Huang & MacKerell, 2013) was used for the protein and adapted for the ligand. TIP3P (Jorgensen et al., 1983) water molecules were added to build a solvation layer of 10-Å minimum thickness. Energy minimization was performed with the steepest descent for 5,000 steps. The particle mesh Ewald method was applied to treat periodic electrostatic interactions using a cutoff distance of 12 Å. The Lennard–Jones potential was smoothed in the range of 10–12 Å. All bonds involving hydrogen atoms were constrained using LINCS (Hess et al., 1997). System equilibration was conducted in the NpT ensemble using the Berendsen barostat and applying a compressibility of 4.5 × 10^−5^ bar^−1^ and a time constant of 1.0 ps. Temperature control was performed using the velocity rescaling method with a stochastic term (Bussi et al., 2007). Since our goal was to relax the conformation of the amino acid residues that were manually modified and not to sample conformations at thermodynamically relevant conditions, the temperature was gradually increased to 90.15 K. The relaxed structure of UGT-23 bound to UDP was used as the receptor for virtual screening, which was performed using AutoDock Vina (Trott and Olson, 2010). The search space was defined as a box encompassing exposed residues in the substrate binding site with 1-Å grid spacing and an exhaustiveness parameter of 20.

## 3 Results

### 3.1 Candidate selection

**Figure 3:**
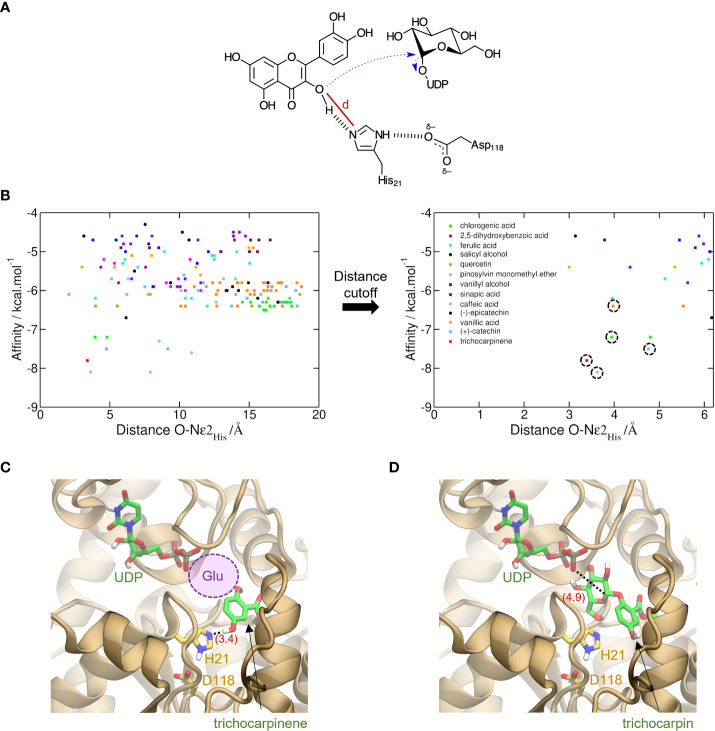
Prediction of activity of trichocarpinene (15) via virtual screening of metabolites against UGT-23. **(A)** Proposed electron flow (dashed blue arrows) in the glycosylation of aromatic metabolites by UGTs. In the example, quercetin (34), bound to UGT-23, interacts with His21, which acts as the general base to initiate the nucleophilic attack at C1′ of glucose-UDP. The cutoff distance *d* used to filter out unproductive conformations generated via molecular docking is depicted with a red line. **(B)** Virtual screening of 17 metabolites against UGT-23. For each of the nine conformations generated for these metabolites, the AutoDock Vina-predicted binding affinity and the cutoff distance *d* are shown with circles and squares of different colors, as labeled. These values are also depicted for the top-ranked conformation of trichocarpinene (red square). In the plot on the right, which shows only results with lower *d* values, the symbols corresponding to the active compounds in UGT-23 are highlighted with a dashed circle, namely, (+)-catechin (37), chlorogenic acid, pinosylvin monomethyl ether (23), quercetin, and trichocarpinene (predicted to be active). Top-ranked structure of trichocarpinene **(C)** and trichocarpin **(D)** bound to UGT-23 (orange cartoon) predicted with AutoDock Vina (Trott & Olson, 2010). The key residues for catalysis, His21 and Asp118, are depicted (yellow carbon, licorice representation). The distances between Nϵ2 in the His21 and the hydroxyl oxygen in 15 and between the phosphatidyl oxygen in the uridine diphosphate (UDP) and C1′ in the glucose-UDP are depicted in parentheses (red). The UDP coordinates were transferred from the aligned structure of UGT78G1 (PDB_ID: 3HBF [Modolo et al., 2009]). The region that would be occupied by a glucosyl moiety attached to the UDP in panel **(C)** is represented with a violet circle.

Analysis of the mGWAS network revealed that 106 hypothetical UGTs had one or more significant mGWAS phenotype associations. Downselection for full-length ORFs and maximum uniqueness resulted in 40 UGT candidates (**Figures 3A, B**; **Tables 1**, **S1**), which were associated with 67 unique metabolites, including benzenoids (specialized metabolites of the Salicaceae family), phenylpropanoids (lignans and flavonoids), and their glycoconjugates (**Figure 3C**; **Table S2**). We calculated one to eight associations per UGT, with a mean of 2.4 ± 1.4.

**Table 1:** *Populus trichocarpa* candidate UGTs, predicted enzymatic activity based on functional annotations, and GWAS-associated metabolites.

UGT ID	Gene Model	UGT Subfamily	Functional Annotation	GWAS Associated Metabolites
UGT-1	Potri.001G030600	UGT91A1-RELATED	soyasaponin III rhamnosyltransferase	**4-hydroxyacetophenone (14)**, benzyl-coumaroyl-glucoside, malic acid
UGT-2	Potri.002G162200		soyasaponin III rhamnosyltransferase	3-O-caffeoyl-quinate
UGT-3	Potri.004G123500		trans-zeatin O-beta-D-glucosyltransferase	fructose, partial_id
UGT-4	Potri.004G214100		trans-zeatin O-beta-D-glucosyltransferase	gentisic acid-2-O-glucoside, glyceric acid
UGT-5	Potri.006G179700		anthocyanidin 3-O-glucosyltransferase	gentisic acid-2-O-glucoside, trichocarpin, partial_id
UGT-6	Potri.007G030500		anthocyanidin 3-O-glucosyltransferase	partial_id
UGT-7	Potri.007G132400		cyanohydrin beta-glucosyltransferase	partial_id
UGT-8	Potri.009G077400		trans-zeatin O-beta-D-glucosyltransferase	2-hydroxyglutaric acid
UGT-9	Potri.009G133300	UGT78D1-RELATED	anthocyanidin/Flavonol 3-O-glucosyltransferase	partial_id
UGT-10	Potri.010G182600		soyasaponin III rhamnosyltransferase	6-hydroxy-2-cyclohexenone-1-carboxylic acid, partial_id
UGT-11	Potri.014G088400		soyasaponin III rhamnosyltransferase	partial_id
UGT-12	Potri.016G016600	UGT71D1-RELATED	anthocyanidin 3-O-glucosyltransferase	threonine, partial_id
UGT-13	Potri.016G017400		anthocyanidin 3-O-glucosyltransferase	partial_id
UGT-14	Potri.016G019400	UGT82A1	trans-zeatin O-beta-D-glucosyltransferase	**secoisolariciresinol**,1,2-cyclohexanediol-glucoside, partial_id
UGT-15	Potri.016G020800	UGT85A24	7-deoxyloganetin glucosyltransferase	**secoisolariciresinol**, partial_id
UGT-16	Potri.016G022000		cyanohydrin beta-glucosyltransferase	**secoisolariciresinol**, partial_id
UGT-17	Potri.016G022100		cyanohydrin beta-glucosyltransferase	**secoisolariciresinol**, partial_id
UGT-18	Potri.016G057300	UGT55-RELATED	trans-zeatin O-beta-D-glucosyltransferase	ribulose, partial_id
UGT-19	Potri.017G042800		soyasaponin III rhamnosyltransferase	Z-9-heptadecosene
UGT-20	Potri.018G008900	UGT90A1-RELATED	flavonol 3-O-glucosyltransferase	partial_id
UGT-21	Potri.018G140400	UGT91A1-RELATED	soyasaponin III rhamnosyltransferase	1,2-cyclohexanediol-glucoside, partial_id
UGT-22	Potri.003G210400		glucosyl/glucuronosyl transferases†	**luteolin**, partial_id
UGT-23	Potri.006G171200		flavonol-3-O-rhamnosyltransferase‡	benzyl-coumaroyl-glucoside, benzyl alcohol, trichocarpin, **trichocarpinene (15)**, partial_id
UGT-24	Potri.007G030400	UGT72E	coniferyl-alcohol glucosyltransferase	stearic acid, tremuloidin conjugate, partial_id
UGT-25	Potri.011G097900	UGT79B1	anthocyanidin 3-O-glucoside 2'''-O-xylosyltransferase	partial_id
UGT-26	Potri.017G052400		7-deoxyloganetin glucosyltransferase	**tyrosol (12)**, 6-hydroxy-2-cyclohexenone-1-carboxylic acid, benzoyl-salicyloylsalicin, **salidroside (13)**, tyrosol-4-O-glucoside
UGT-27	Potri.002G236400	UGT75C1	anthocyanidin 3-O-glucoside 5-O-glucosyltransferase	partial_id
UGT-28	Potri.004G119700	UGT83A1	UDP-glycosyltransferase 83A1	tremulacin, partial_id
UGT-29	Potri.006G022500	UGT85A24	7-deoxyloganetin glucosyltransferase	benzyl alcohol, partial_id
UGT-30	Potri.006G055600		crocetin glucosyltransferase	partial_id
UGT-31	Potri.009G095500		(indol-3-yl)acetate beta-D-glucosyltransferase§	fructose, **gallic acid (8)**
UGT-32	Potri.013G143900	UGT78D1-RELATED	anthocyanidin/Flavonol 3-O-glucosyltransferase	salicyltremuloidin, partial_id
UGT-33	Potri.014G175000	UGT74B1	salicylic acid glucosyltransferase§	**dihydromyricetin**, galactinol, salicyltremuloidin, partial_id
UGT-34	Potri.015G071900	UGT74B1	N-hydroxythioamide S-beta-glucosyltransferase	**dihydroquercetin**, partial_id
UGT-35	Potri.016G014100	UGT71B2-RELATED	similar to hypostatin glucosyltransferase¶	catechol, partial_id
UGT-36	Potri.016G016100	UGT71D1-RELATED	UDP-glycosyltransferase 71D1-related†	partial_id
UGT-37	Potri.016G016800	UGT71D1-RELATED	similar to hypostatin glucosyltransferase¶	threonine, partial_id
UGT-38	Potri.017G032700	UGT74D1	salicylic acid glucosyltransferase§	tyrosol-4-O-glucoside, partial_id
UGT-39	Potri.017G077800		glucosyl/glucuronosyl transferases†	**tyrosol (12)**l, (Z,Z, Z)-9,12,15-octadecatrienoic acid, ethyl ester, 5-oxo-proline
UGT-40	Potri.018G096000		anthocyanidin/Flavonol 3-O-glucosyltransferase	partial_id

UGT IDs were arbitrarily assigned and specific to this project. Predicted UGT subfamily identifiers, where available, were obtained from Phytozome. Functional annotations were based on Enzyme Classification (EC) Numbers except where otherwise indicated:

^†^Phytozome, ^‡^MapMan, ^§^KEGG Orthology, ¶Arabidopsis Best Hit Ortholog. Metabolites in bold were selected for enzyme assays. Association with one or more partially identified metabolites is indicated by “partial_id”; for a full list and further details, see **Table S2**.

GWAS, genome-wide association study.

### 3.2 Functional annotation

UGTs were annotated with 10 Enzyme Classification (EC) Numbers (**Figure S1**), seven Kyoto Encyclopedia of Genes and Genomes (KEGG) Orthology (KO) identifiers, and five MapMan Bins (**Figure S3**, **Tables 1**, **S2**). All candidates shared the following annotations: Pfam PF00201, GO terms GO:0008152 and GO:0016758, KOG classification KOG1192, and Panther Family PTHR11926. No additional Pfam domains were present among candidates, strongly suggesting that the genes encode UGTs.

### 3.3 Gene transcription

UGTs in the Gene Atlas heatmap dendrogram (**Figure S2**) formed four major clusters exhibiting the following expression patterns (top to bottom): broad expression across tissues and developmental stages (e.g., UGT-23), highest expression in mature leaf tissues (e.g., UGT-32), highest expression in root tissue (e.g., UGT-1), and highest expression in actively growing tissue (buds, young leaves, and root tips; e.g., UGT-11). Two minor clusters exhibited the highest expression in dormant buds (UGT-28 and UGT-33) and stem tissues (e.g., UGT-39). These results indicate that some candidates exhibit tissue and developmental stage specificity, while others are ubiquitously expressed.

In population RNA-seq analyses, expression varied significantly among UGTs, across the population, and within and across tissues (**Figures S4–S6**). For example, UGT-18 expression was variable in root tissue, but low or absent in leaf and xylem tissue, whereas UGT-35 exhibited relatively high expression across the population in all three tissues. In some cases, tissue-specific patterns in the Gene Atlas analysis were consistent with population-scale analyses.

Overall, UGTs were broadly expressed across the common garden population with some exhibiting tissue-specific transcription, but tissue-specific patterns in the Gene Atlas analysis could not be generalized across the population. Although possibly due in part to a technical error (i.e., RNA-seq read mapping issues given the high identity of many UGTs), the timing of tissue collections, or fine-scale differences in developmental stages among samples, observed differences likely have a genetic basis. More work is needed to understand the specific conditions under which transcription occurs.

### 3.4 Validation of mGWAS associations

Nine commercially available substrates in the mGWAS network were assayed against all candidate UGTs: tyrosol (12), salidroside (13), 4-hydroxyacetophenone (14), luteolin (33), dihydroquercetin (35), dihydromyricetin (36), (−)-secoisolariciresinol (42), gallic acid (8), and trichocarpinene (15) (**Figure S7**; **Table 2**). The biochemical assays were designed to be high throughput, using lysates containing overexpressed UGTs combined with each substrate. An autosampler connected to an LC-MS/MS, coupled with automated data processing using MRM of known substrates and products, improved assay analysis time.

**Table 2:** Observed monoglycosylation of substrates by UGTs *in vitro*.

	Substrate	Gallic Acid	4-(2-hydroxyethyl)phenol	4-Hydroxyacetophenone	Trichocarpinene	Luteolin	Dihydroquercetin	Dihydromyrcetin	Secoisolariciresinol	Salidroside
	% UGTs processing substrate	50%	8%	53%	33%	60%	60%	5%	63%	45%
**UGT ID**	**UGT-1**	1	0	1	0	0	0	0	0	0
	**UGT-2**	1	0	0	0	0	0	0	0	0
	**UGT-3**	0	0	0	0	1	0	0	1	0
	**UGT-4**	0	0	0	0	0	0	0	0	0
	**UGT-5**	0	0	0	0	1	0	0	0	0
	**UGT-6**	1	0	0	0	0	0	0	1	0
	**UGT-7**	1	0	0	0	0	0	0	0	0
	**UGT-8**	1	0	1	0	0	1	0	0	0
	**UGT-9**	1	0	0	0	0	1	0	1	0
	**UGT-10**	0	0	0	0	1	0	0	0	0
	**UGT-11**	ND	0	0	ND	0	1	ND	0	0
	**UGT-12**	0	0	0	0	0	0	0	0	0
	**UGT-13**	1	0	1	1	1	1	0	1	1
	**UGT-14**	1	0	0	0	1	0	0	1	0
	**UGT-15**	ND	0	0	ND	0	0	ND	1	0
	**UGT-16**	0	0	1	1	1	0	0	1	0
	**UGT-17**	0	0	1	0	1	1	0	1	0
	**UGT-18**	0	1	1	0	0	1	0	1	0
	**UGT-19**	ND	0	0	ND	0	1	ND	0	0
	**UGT-20**	1	0	1	1	1	1	0	1	0
	**UGT-21**	1	0	0	0	0	1	0	1	0
	**UGT-22**	1	0	1	1	0	1	0	1	0
	**UGT-23**	1	0	1	1	0	0	0	1	1
	**UGT-24**	1	0	1	1	1	1	0	1	1
	**UGT-25**	0	0	0	1	1	1	0	1	0
	**UGT-26**	0	1	1	0	1	1	0	1	1
	**UGT-27**	0	0	1	1	1	1	0	0	1
	**UGT-28**	1	0	0	0	1	1	0	1	1
	**UGT-29**	1	0	1	1	1	1	0	1	1
	**UGT-30**	1	0	1	0	1	1	1	1	1
	**UGT-31**	0	0	0	0	1	1	0	0	1
	**UGT-32**	0	0	0	0	0	0	0	1	1
	**UGT-33**	1	0	1	1	1	1	0	0	1
	**UGT-34**	0	0	1	0	1	0	0	0	1
	**UGT-35**	0	0	1	0	1	1	0	1	1
	**UGT-36**	1	0	1	1	1	1	0	1	1
	**UGT-37**	1	0	1	1	1	0	0	1	1
	**UGT-38**	0	1	1	1	1	1	1	1	1
	**UGT-39**	1	0	0	0	1	1	0	1	1
	**UGT-40**	0	0	1	0	1	1	0	0	1

Of 13 predicted metabolite–UGT associations, nine were supported based on MS detection of the monoglycosylated product (**Figure 3C**). Dihydromyricetin was accepted by the fewest and secoisolariciresinol was glycosylated by the greatest number of UGTs (2 *vs.* 25). Except for UGT-4 and UGT-12, all enzymes screened in the high-throughput assay were confirmed to have glycosyltransferase activity under the conditions tested. No single enzyme, however, was able to glycosylate all nine of these substrates.

As UGTs are known to produce metabolites with multiple glucose moieties, MS data were analyzed for evidence of multiple glycosylations (**Table S3**). Some reactions (e.g., UGT-31 and gallic acid) only produced multiply glycosylated products, while others (e.g., UGT-23 and trichocarpinene) contained products in multiple glycosylation states. Altogether, enzymatic assays confirmed 10 of 13 predicted metabolite–UGT relationships when considering multiple glycosylation states.

### 3.5 Survey of accepted substrate classes

Forty additional metabolites, which were selected based on structural similarity to the original nine compounds or to the backbones of other uncharacterized network metabolites and because of their roles in plant stress response or cell wall biosynthesis, were tested as substrates for glycosylation (**Figure S7**). Nearly all metabolites tested were monoglycosylated by a subset of UGTs (**Tables S3**, **S4**; **Figure S8**), with an average of 15 ± 10 (38% ± 25%) UGTs processing each substrate. Of the 49 total substrates tested, none were glycosylated by every UGT, although quercetin (34) was processed by the greatest percentage (90% or 36 UGTs). Although coumarins were, on average, monoglycosylated by more UGTs than other classes (49% ± 14%) and phenylpropanoids by the fewest (30% ± 24%), differences between metabolite classes were not significant (*p* > 0.05) (**Figure S8**).

For most substrates, multiple glycosylation states were observed, such as in the case of trichocarpinene, for which both mono- and diglycosylated products were observed (**Table S3**). Monoglycosylated indole-3-acetic acid (43; IAA) was not detected in any reaction, but 11 total unique UGTs were able to di-, tri-, or tetra-glycosylate the phytohormone. In the case of salidroside, 13 UGTs had activity when expanding the search criteria to include the diglucoside, although 11 of 18 UGTs with detectable monoglycosylated product were not able to produce the diglucoside under the assay conditions.

UGTs with shared metabolite profiles were compared using a phylogenetic tree (**Figure 3A**; **Table S4**). No obvious relationship between gene sequence similarity and substrate acceptance profile was noted, reinforcing the notion that substrate specificity and enzymatic activity cannot be simply deduced or predicted from the amino acid sequence.

### 3.6 UGT-23 docking analysis

AutoDock Vina-predicted binding affinities (Trott and Olson, 2010) were used to rank order substrates using nine conformations for each metabolite. Cutoff distance, defined based on the likely reaction mechanism, was used to filter out bad poses (Shao et al., 2005). With the use of UGT-23 as a representative UGT, catalytic residues were identified: His21 acts as the general base that potentializes aromatic hydroxyl oxygen in the substrate, which causes the nucleophile to react with C1′ of glucose-UDP (**Figure 2A**). Interaction of His21 with Asp118 assists in the initialization of electron transfer.

The distance cutoff for best docking poses was 5.5 Å between the aromatic hydroxyl oxygens in the glucose acceptor and Nϵ2 in His21. Within the distance cutoff, pinosylvin monomethyl ether (23), trichocarpinene, (+)-catechin (37), chlorogenic acid (24), and quercetin had the lowest predicted binding affinity values (i.e., strongest binders) among known active compounds of UGT-23 (**Figure 2B**). Molecular docking of trichocarpin, the glucoside of trichocarpinene, and a predicted substrate of UGT-23 (**Figure 3D**) predicted a top-ranked conformation in the binding site that is appropriately oriented as the product of the glycosylation of trichocarpinene (**Figure 2C**), confirming *in vitro* assay results that demonstrated that UGT-23 glycosylates trichocarpinene.

## 4 Discussion

High-throughput biochemical assays were used to validate an mGWAS network. Forty UGT genes were selected from the merged mGWAS network based on sequence diversity and the likelihood that candidates encoded unique functional UDP-glycosyltransferases. RNA-seq analyses confirmed that all candidates exhibited expression, and functional annotation strongly indicated that glycosylation is the native role of these enzymes in *P. trichocarpa*.

All UGTs were active on five or more substrates (**Table S3**). Substrate scope varied from five (UGT-7, UGT-12, and UGT-15) to 38 (UGT-36) with a mean of 18 ± 10 (38% ± 20%) monoglycosylated products (**Table S3**). The nine substrates from the mGWAS network were glycosylated by more UGTs than the network predicted, suggesting relaxed substrate specificity in our assays. Substrates included benzenoids, phenylpropanoids, phytohormones, and terpenoids, which function in development, defense response, cell wall biosynthesis, pigmentation, and creation of aromatic scents (Le Roy et al., 2016). No single substrate class was significantly glycosylated compared to other classes, suggesting *P. trichocarpa* UGTs have evolved to process a wide variety of metabolites. Also, differences in glycosylation patterns of structurally similar compounds suggest that the UGTs have different requirements for substrate structure, or that other factors, such as co-localization within the plant or expression conditions, may be involved in substrate acceptance. Protein phylogeny was not correlated with the number or type of substrates processed, which indicates that nuanced differences in the binding pocket structure, and not protein sequence alone, may dictate substrate specificity.

Quercetin was the most widely accepted substrate, with 36 of 40 UGTs producing quercetin glucoside. This substrate was selected because it is structurally similar to dihydroquercetin, an antifungal flavonoid identified in the mGWAS network, and because a quercetin conjugate of unknown structure was associated with UGTs 13–17 in the mGWAS network (**Figure 3C**). While only two of these five enzymes glycosylated dihydroquercetin, four glycosylated quercetin, highlighting the differences in substrate acceptance of highly similar molecules. These enzymes may also glycosylate the quercetin conjugate, although metabolite isolation, structure elucidation, and testing are needed for confirmation.

Interestingly, none of the enzymes produced IAA-monoglucoside; only multiply glycosylated IAA was detected in UGT reactions (**Table S3**). IAA is an important phytohormone in plant growth and development, and it serves as a quorum sensing or communication molecule in bacteria (Estenson et al., 2018). IAA gluco-conjugates are thought to form as a way to remove excess signaling molecules (Yamaguchi et al., 2010). Multiple glucose moieties are common in natural product biosynthesis and can trigger different response pathways in the plant (Thibodeaux et al., 2007; Williams et al., 2008). Tests to identify which glycoconjugate is the major product of each UGT-substrate pair could provide additional insight into the relative weights of relationships in the metabolite-gene network.

Trichocarpinene and trichocarpin, which differ by a glucose moiety, were associated with UGT-23 in the mGWAS network (**Figure 3C**). While trichocarpin was not directly tested as a substrate, both mono- and diglycosylated products of trichocarpinene were observed. Virtual screening of active compounds with UGT-23 indicated that trichocarpinene has a strong binding affinity compared to other substrates and that trichocarpin is the product of the enzyme-catalyzed glycosylation of trichocarpinene, rationalizing the predicted and experimentally validated association between trichocarpin and UGT-23.

Like previous studies (Chen et al., 2020; Kurze et al., 2022; Salas and Mendez, 2007; Zhang et al., 2006), we observed relaxed substrate acceptance profiles, suggesting that *P. trichocarpa* UGTs are not specific for particular substrates. However, biochemical assays often over- or under-represent *in vivo* enzyme activity, depending on reaction conditions. The high-throughput design of this workflow prevented extensive testing of various conditions, which could reveal nuances in associations and activities. This may also explain why three metabolite-gene associations were not reflected in enzyme assays. Assaying substrate processing under alternate reaction conditions (e.g., additives or effectors in the reaction mixture, and different reaction temperatures and times) would further inform predicted metabolite-gene relationships and substrate specificity, and incorporating kinetic assays and substrate competition assays into the workflow would reveal preferred substrates (Wang et al., 2019; Tan et al., 2022). Furthermore, investigating the variability in tissue-specific UGT activity reflected in the RNA-seq analyses, as well as determining the subcellular location of glycosylation reactions, would provide valuable information regarding *in vivo* specificity. Another limitation of our approach is that it is unlikely to identify the function of enzymes involved in protein–protein interactions or heterocomplexes (e.g., Maszczak-Seneczko et al., 2012). Furthermore, the workflow is limited to enzymes that can be expressed in a heterologous host and to metabolites that have been positively identified and are commercially available or easily synthesized.

Nevertheless, this functional genomics workflow enabled the characterization of the substrate acceptance of a set of enzymes, and it also validated predicted associations in an mGWAS network. As biologists increasingly turn to predictive models to study metabolite-gene associations, reliable methods to test the models are necessary. Such validation studies can aid in improving functional genomics models and identify gene targets for manipulating metabolite production.

In conclusion, an increasing number of studies leverage whole genome sequencing data in combination with high-throughput phenotyping to identify candidate genes (Chen et al., 2020; Rehman et al., 2020; Wei et al., 2021; Li et al., 2001; Wu et al., 2016). However, downstream functional characterization remains a bottleneck: most GWAS-led studies validate two or fewer candidates, with many studies omitting validation altogether. Here, we demonstrated the use of mGWASs to prioritize significant metabolite-gene associations, which we validated using high-throughput biochemical assays. The identified functional *P. trichocarpa* UGTs are putatively involved in lignan, flavonoid, salicylate, and phytohormone metabolism with implications for cell wall biosynthesis, nitrogen uptake, and biotic and abiotic stress responses that determine sustainable biomass crop production. Our results provide direction for further *in vitro* and *in vivo* functional characterization in which enzyme activities and downstream effects can be interrogated. These studies have implications for identifying enzymes that can transform secondary metabolites with utility in biomedical and bioenergy applications.

## Licenses and permissions

This manuscript has been authored by UT-Battelle, LLC, under contract DE-AC05-00OR22725 with the US Department of Energy (DOE). The US government retains and the publisher, by accepting the article for publication, acknowledges that the US government retains a nonexclusive, paid-up, irrevocable, worldwide license to publish or reproduce the published form of this manuscript, or allow others to do so, for US government purposes. DOE will provide public access to these results of federally sponsored research in accordance with the DOE Public Access Plan (http://energy.gov/downloads/doe-public-access-plan).

## Data availability statement

The *P. trichocarpa* genome sequence, annotation data, and Gene Atlas expression data are available at https://phytozomenext.jgi.doe.gov/. *P. trichocarpa* SNP andindel data are available at https://cbi.ornl.gov/gwas-dataset/. *P. trichocarpa* leaf, xylem, and root RNA-seq are available in the NCBI SRA database (www.ncbi.nlm.nih.gov/sra; SRP097016-SRP097036). The accession numbers of the UGTs in this study are provided in thesupplemental materials. The data obtained from high-throughput MS-based analysis of enzyme assays are in the **Supplemental Materials**.

## Author contributions

PS-V: methodology, validation, formal analysis, investigation, data curation, writing—original draft, writing—review and editing, and visualization. AF: software, formal analysis, investigation, data curation, writing—original draft, writing—review and editing, visualization, and project administration. SG: conceptualization, methodology, validation, formal analysis, investigation, resources, data curation, writing—original draft, supervision, project administration, and funding acquisition. EP: formal analysis, investigation, data curation, writing—original draft, writing—review and editing, and visualization. PJ: formal analysis, data curation, and writing—review and editing. JA: formal analysis and investigation. AL: formal analysis, and investigation. NZ: formal analysis and investigation. MM: formal analysis and investigation. PR: formal analysis and investigation. DK: formal analysis, data curation, and writing—review and editing. UK: resources, data curation, and writing—review and editing. J-GC: resources, data curation, writing—review and editing. WM: formal analysis, investigation, data curation, writing—original draft, and writing—review and editing. DJ: conceptualization, resources, data curation, writing—review and editing, supervision, and funding acquisition. TT: conceptualization, methodology, resources, data curation, writing—original draft, writing—review and editing, supervision, project administration, and funding acquisition. All authors contributed to the article and approved the submitted version.
