# Supplementary material for: Validation of a metabolite–GWAS network for Populus trichocarpa family 1 UDP-glycosyltransferases
Source: Front Plant Sci. 2023 Jul 21;14:1210146. doi: 10.3389/fpls.2023.1210146 (PMC10402742; doi:10.3389/fpls.2023.1210146)
Supplement: Supplementary file 1 [file DataSheet_1.docx]

***Supplementary Material***

**Validation of a metabolite-GWAS network for *Populus trichocarpa* family 1 UDP-glycosyltransferases**

**Patricia M. B. Saint-Vincent*, Anna Furches*, Stephanie Galanie, Erica Teixeira Prates, Jessa L. Aldridge, Audrey Labbe, Nan Zhao, Madhavi Z. Martin, Priya Ranjan, Piet Jones, David Kainer, Udaya C. Kalluri, Jin-Gui Chen, Wellington Muchero, Daniel Jacobson, Timothy J. Tschaplinski†**

*** Correspondence:** Timothy Tschaplinski: tschaplinstj@ornl.gov

**Figure S1.** UGT candidate gene set Enzyme Classification (EC) annotations.

**Figure S2.** Gene Atlas clustered heatmap for 40 UGT candidate genes showing transcription in four tissues over multiple developmental stages in the *Populus trichocarpa* reference, Nisqually-1.

**Figure S3.** UGT candidate gene set **(A)** KEGG Orthology (KO) and **(B)** Mapman annotations.

**Figure S4.** Clustered heatmap of GWAS population leaf tissue RNA-seq data for UGT candidates.

**Figure S5.** Clustered heatmap of GWAS population root tissue RNA-seq data for UGT candidates.

**Figure S6.** Clustered heatmap of GWAS population xylem tissue RNA-seq data for UGT candidates.

**Figure S7.** Compounds tested as substrates for the *P. trichocarpa* UGTs. Classes of similar substrates are inscribed in boxes.

**Figure S8.** Percent of UGTs that produced a monoglucoside **(A)** and average acceptance rate by substrate class **(B)**. Error bars indicate standard deviation of the mean for each class of structurally related compounds.
